# Impact of Dietary Isoflavone Supplementation on the Fecal Microbiota and Its Metabolites in Postmenopausal Women

**DOI:** 10.3390/ijerph18157939

**Published:** 2021-07-27

**Authors:** Lucía Guadamuro, M. Andrea Azcárate-Peril, Rafael Tojo, Baltasar Mayo, Susana Delgado

**Affiliations:** 1 Instituto de Productos Lácteos de Asturias (IPLA-CSIC), Departament of Microbiology and Biochemistry of Dairy Products, Paseo Río Linares s/n, 33300 Villaviciosa, Spain; luciaguadamurogarcia@gmail.com (L.G.); baltasar.mayo@ipla.csic.es (B.M.); 2 Division of Gastroenterology and Hepatology, and Microbiome Core, School of Medicine, Department of Medicine, University of North Carolina (UNC), Chapel Hill, NC 2759, USA; azcarate@med.unc.edu; 3 Gastroenterology Department, Cabueñes University Hospital, 33203 Gijón, Spain; tojorafael@uniovi.es; 4 Instituto de Investigación Sanitaria del Principado de Asturias (ISPA), Avenida de Roma s/n, 33011 Oviedo, Spain

**Keywords:** fecal microbiota, isoflavones, equol, pyrosequencing, menopause, fatty acids

## Abstract

Isoflavones are metabolized by components of the gut microbiota and can also modulate their composition and/or activity. This study aimed to analyze the modifications of the fecal microbial populations and their metabolites in menopausal women under dietary treatment with soy isoflavones for one month. Based on the level of urinary equol, the women had been stratified previously as equol-producers (*n* = 3) or as equol non-producers (*n* = 5). The composition of the fecal microbiota was assessed by high-throughput sequencing of 16S rRNA gene amplicons and the changes in fatty acid excretion in feces were analyzed by gas chromatography. A greater proportion of sequence reads of the genus *Slackia* was detected after isoflavone supplementation. Sequences of members of the family *Lachnospiraceae* and the genus *Pseudoflavonifractor* were significantly increased in samples from equol-producing women. Multivariable analysis showed that, after isoflavone treatment, the fecal microbial communities of equol producers were more like each other. Isoflavone supplementation increased the production of caproic acid, suggesting differential microbial activity, leading to a high fecal excretion of this compound. However, differences between equol producers and non-producers were not scored. These results may contribute to characterizing the modulating effect of isoflavones on the gut microbiota, which could lead to unravelling of their beneficial health effects.

## 1. Introduction

The existence of an inter-individual variability in response to diet and lifestyle interventions is widely accepted [1]. A complex interaction between diet, human genome, and the gut microbiome occurs and can determine the effects of dietary bioactives [2]. The gut microbiota is a critical component that can alter the absorption and metabolism of foods, and thus the final effects on human health. However, although a growing body of studies exists, the mechanisms underlying these processes are complex and not entirely understood. In this context, isoflavones-plant-derived polyphenols found at a relatively high concentration in soy and soy-derived products have been related to diverse health benefits such as the prevention of chronic diseases, including hormone dependent cancer, cardiovascular diseases, osteoporosis, and postmenopausal syndrome [3]. Although there is scientific evidence of the beneficial effects in counteracting symptoms like hot flushes and vasomotor reactions in menopausal women [4], the European Food Safety Authority (EFSA) has refuted health claims about the role of isoflavones in body functions [5]. The clinical effectiveness of ingested isoflavones might be due to their ability to be converted into active metabolites like equol [6,7]. This metabolite is the isoflavone-derived compound with the strongest estrogenic activity and antioxidant capacity, and is generated by specific members of the gut microbiota. Only some individuals harbor the microbiota required for the conversion into equol, resulting in different metabotypes: equol producers and non-producers [8]. Remarkably, compared with that in Asian populations (50–60%), the equol producer metabotype has a prevalence of 25–30% in Western populations [7].

Although the full range of intestinal bacteria involved in equol formation remains unknown [8,9], most of the equol-producing bacteria characterized so far are members of the family *Coriobacteriaceae* [10]. Additionally, the microorganisms responsible for equol production might differ across individuals [11,12,13].

Like other polyphenols, isoflavones are metabolized by components of the microbiota, and at the same time, they could also modulate the composition and/or activity of the intestinal microbial populations [14]. Analysis of intestinal microbiota modifications after isoflavone consumption could give clues as to the microorganisms involved in its metabolism. Some studies analyzed the effects of the isoflavone intake on the gut microbiota [15,16,17,18,19]. However, additional studies applying high-throughput approaches are still needed to determine low abundance microorganisms, like those probably involved in equol production.

This study aimed to determine changes in the intestinal microbiota induced by a 1-month period of isoflavone consumption and to explore changes related to the equol status metabotype. With this aim, high-throughput amplicon sequencing of the bacterial 16S rRNA gene was performed on fecal samples taken before and after isoflavone consumption by eight menopausal women (three equol producers and five equol non-producers). In addition, metabolite analysis of feces was performed using gas chromatography for determination of possible shifts in fatty acid excretion.

## 2. Materials and Methods

### 2.1. Human Volunteers

Ethical approval for this study was obtained from the Bioethics Subcommittee of the Spanish Research Council (Consejo Superior de Investigaciones Científicas or CSIC) and the Regional Ethics Committee for Clinical Research of the Health Service of Asturias (Servicio de Salud del Principado de Asturias) (approval number: 15/2011), in compliance with the Declaration of Helsinki. Fecal samples were provided, with written consent, by eight postmenopausal women recruited during a preceding study [18] at the Gynecology and Obstetrics Unit (in collaboration with the Gastroenterology Department) of Cabueñes Hospital (Gijón, Spain). The participants did not suffer from any infectious diseases or intestinal disorder. Additionally, they had not received antibiotics or any other medication for at least 6 months prior to the collection of samples. The women had been identified with an equol-producing metabotype (or not), based on the levels of urinary equol excretion [20]. For the present work, we selected three of the women (WC, WG, and WP) with an equol-producing phenotype (urine equol > 1000 nM as defined by Rowland et al. [21]) and five women (WE, WH, WF, WL, and WN) with a non-producing phenotype (excreted equol in urine ranging from 0 to 377 nM). Participants reported consuming a normo-type, Mediterranean diet and did not start following a vegetarian, vegan, or special diet during the intervention period. Supplementation consisted of a daily oral intake (80 mg/day) in the morning of a commercial dietary supplement (Fisiogen; Zambon, Bresso, Italy) rich in soy isoflavones (55–72% genistin/genistein, 28–45% other isoflavones) for one month.

### 2.2. Sample Collection

The study was conducted during the fall–winter seasons of 2011–2012. The volunteers provided samples of feces before treatment (basal, T0) and after one month (T1) of isoflavone supplementation. Fresh stools were collected in sterile plastic containers and kept under anaerobic conditions in jars containing Anaerocult A (Merck, Darmstadt, Germany) for transporting to the laboratory within 2 h. Fecal samples were kept frozen at −80 °C until use.

### 2.3. Total Bacterial DNA Extraction

Fecal samples (0.2 g) were suspended in 1.8 mL of phosphate buffered saline (PBS) (pH 7.4). These suspensions were homogenized and centrifuged at 800 rpm for 5 min at 4 °C to eliminate insoluble material, and the supernatants were transferred to new tubes. These were then centrifuged again at 14,000 rpm for 5 min at 4 °C. Pelleted cells were suspended in 1 mL of PBS and lysed in an enzyme solution containing 20 mM TRIS-HCl pH 8.0, 2 mM EDTA, 1.20% Triton X-100, 20 mg/mL lysozyme (Merck), and 20 U mutanolysin (Sigma-Aldrich, Saint Louis, MO, USA). Total bacterial DNA was extracted following the protocol described by Zoetendal [22], and purified using the QIAamp DNA Stool Minikit (Qiagen, Hilden, Germany). Finally, the DNA was eluted in 100 µL of sterile molecular grade water (Sigma-Aldrich), and its concentration and quality were determined using an Epoch microvolume spectrophotometer (BioTek Instruments, Winooski, VT, USA).

### 2.4. Library Construction and Pyrosequencing

A segment of the 16S rRNA genes from the purified bacterial DNA were PCR-amplified using the universal primers Y1 (5′-TGGCTCAGGACGAACGCTGGCGGC-3′) (position 20–43 on the 16S rRNA gene of Escherichia coli) and Y2 (5′-CCTACTGCTGCCTCCCGTAGGAGT-3′) (positions 361–338). These primers amplify a 348 bp stretch of the prokaryotic rDNA embracing the V1 and V2 hypervariable regions. Further, 454-adaptors were included in both the forward (5′-CGTATCGCCTCCCTCGCGCCATCAG-3′) and reverse (5′-CTATGCGCCTTGCCAGCCCGCTCAG-3′) primers, followed by a 10 bp sample-specific barcode. Amplifications were performed using the NEBNext High-Fidelity 2x PCR Master Mix Kit (New England Biolabs., Ipswich, MA, USA) as follows: 95 °C for 5 min, 25 cycles of 94 °C for 30 s, 60 °C for 45 s, 72 °C for 30 s, and a final extension step at 72 °C for 5 min. The amplicons produced were purified using the GenElute PCR Clean-Up Kit (Sigma-Aldrich), and their concentration was measured in a Qubit fluorometer with dsDNA assay kits (Thermo Fisher Scientific Inc., Waltham, MA, USA).

An amplicon library was prepared for pyrosequencing by mixing equal amounts of amplicons from the different samples. Pooled amplicons were then sequenced using a 1/8 picotitre plate in a 454 Titanium Genome Sequencer (Roche, Indianapolis, IN, USA) in the UNC Microbiome Core (University of North Carolina, USA).

### 2.5. Sequence and Data Analysis

Raw sequences were denoised and filtered out of the original dataset. Filtering and trimming were performed using the Galaxy Web Server [23], employing the sliding window method. Only reads longer than 150 bp were used in further analysis. Chimeras were eliminated using the USEARCH v.6.0.307 clustering algorithm routine in de novo mode [24]. After demultiplexing, high quality rDNA sequences were classified taxonomically using the Ribosomal Database Project (RDP) Bayesian Classifier [25] with an 80% confidence threshold to obtain the taxonomic assignment and relative abundance of the different bacterial groups. “Genus” was the lowest taxonomic level contemplated. Sequences with at least 97% similarity were clustered into operational taxonomic units using the CD-Hit clustering method [26] and employed in the generation of rarefaction curves using a RarefactWin freeware (produced by S. Holland; http://strata.uga.edu/software/index.html). Different diversity indexes (Sobs, Chao, ACE, Jackknife, Shannon, Simpson) were calculated for each sample and compared between groups of women [27]. As diversity index values increase with sample size, normalization of sequencing effort in all samples was necessary to avoid biases in the results [28]. Thus, diversity indexes were normalized using the median number of sequences obtained in all samples as a scaling factor [29]. Weighted UniFrac analysis [30] was performed to assess the similarity of the microbial communities between samples and principal coordinates analysis (PCoA) was applied to the distance matrix for visualization.

### 2.6. Fatty Acids (FAs) Determination

One hundred microliters of a 1:10 dilution of feces (*w*/*v*) in PBS was supplemented with 100 μL of 2-ethyl butyric acid (Sigma-Aldrich, St. Louis, MO, USA) as an internal standard (1 mg/mL in methanol) and acidified with 100 μL of 20% formic acid (*v/v*). The acidic solution was then extracted with 1 mL of methanol and centrifuged for 10 min at 15,700× *g*. Supernatants were kept at −20 °C until analysis in a 6890 N gas chromatography (GC) apparatus (Agilent Technologies, Santa Clara, CA, USA) connected to a flame ionization detector (FID). All samples were analyzed in duplicate and FAs were quantified as previously described [31].

### 2.7. Statistical Analysis

Statistical analysis of data was performed using IBM SPSS 23 statistic software. The Mann–Whitney test for independent samples was performed to examine differences between equol producers and non-producers in terms of microbial groups, diversity indexes, and fecal FAs. The Wilcoxon signed-rank test for related samples was used to examine differences between before and after isoflavone supplementation. Alternatively, Student’s *t*-test was used when normal distribution was confirmed using Saphiro-Wilk test. Two-tailed probability values of *p* < 0.05 were considered significant.

## 3. Results

### 3.1. Change in Fecal Microbiota Over Isoflavone Supplementation

After denoising, performing chimera checks, and trimming the reads by length (150–400 bp), a mean of 4756 (±875) high quality sequences was obtained. Taxonomic analysis grouped the sequences mainly into five phyla: *Firmicutes, Actinobacteria, Bacteroidetes, Proteobacteria*, and *Verrucomicrobia*. Fifty-two genera were identified, as well as five groups of clostridia (*Clostridium* cluster IV, cluster XI, cluster XIVa, cluster XVIII, and *Clostridium sensu stricto*) and two taxa with family-associated *incertae sedis* (*inc. sed.*) members (*Erysipelotrichaceae inc. sed.* and *Lachnospiraceae inc. sed.*). Taxonomic groups presenting at an abundance of <0.1% were designated as “others”. A mean of 1813 sequences per sample remained unclassified.

**Figure 1:**
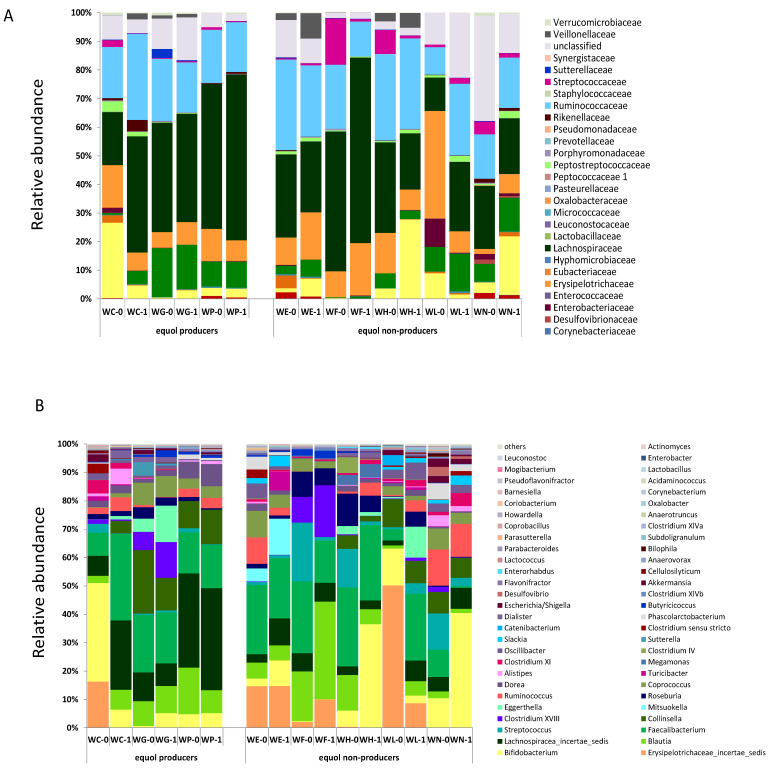
Changes in microbial composition with isoflavone supplementation. Microbial composition at the family (**A**) and genus (**B**) levels in fecal samples of eight menopausal women before (T0) and after one month (T1) of isoflavone supplementation presented as relative abundances (%).

Considerable differences were observed between the bacterial communities at T0 (before isoflavone supplementation) and T1 (one month after supplementation). Differences were noted at the family and genus levels (Figure 1). At the genus level, a significant (*p* < 0.05) increase in the relative abundance of the genus *Slackia* was observed at T1 (0.67%) versus T0 (0.27%). Although sequences of this genus were not detected in all women, when they were detected (WC, WG, WE, WL, and WN), their relative proportion increased after supplementation with soy isoflavones.

**Figure 2:**
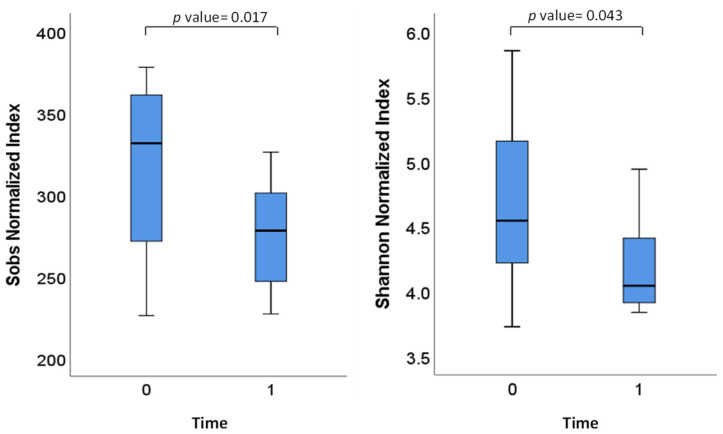
Comparison of Sobs and Shannon indexes before (T0) and after one month (T1) of isoflavone treatment in eight menopausal women. The lines inside the rectangles indicate the medians and the whiskers indicate the maximum and minimum values. Analysis was done using the nonparametric Wilcoxon signed-rank test to determine differences between T0 and T1.

The supplementation with isoflavones significantly reduced alpha diversity in terms of Sobs and Shannon Indexes (Figure 2). The Sobs index reflects the number of observed species or “richness”, while Shannon index weights the numbers of species by their relative evenness.

### 3.2. Differences in Microbial Groups Associated with the Equol Producer Status

**Figure 3:**
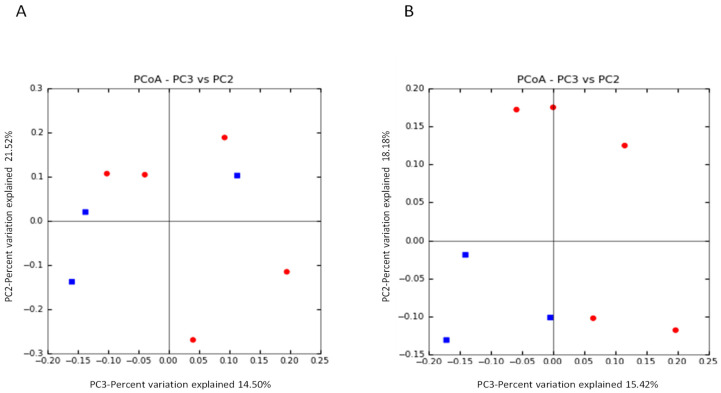
Weighted UniFrac principal coordinates analysis (PCoA) plots of fecal microbiota composition from the women in the study (*n* = 8) before soy isoflavone intervention (**A**), and after one month of daily supplementation (**B**). Subject color coding: red, equol non-producers (*n* = 5); blue, equol producers (*n* = 3).

UniFrac β-diversity analysis was done to assess the extent of similarity between microbial communities. UniFrac-based PCoA plots revealed a clear clustering between equol producing and non-producing women after isoflavone supplementation, while no clustering was observed at baseline (Figure 3).

Furthermore, comparison of fecal microbial composition between equol producers and non-producers revealed some differences. At T0, relative abundance (% sequences) of *Lachnospiraceae inc. sed.* taxa was significantly higher (*p* = 0.025) in the equol-producer group versus the non-producers (Table 1). While at T1, after one month of isoflavone consumption, the relative abundance of sequences belonging to the genera *Pseudoflavonifractor* and *Dorea* was greater in the equol-producing women.

### 3.3. Differences in Fatty Acids (FAs) Associated with the Equol Producer Status

Fecal FAs remained stable after 1 month of isoflavone supplementation, except for caproic acid, which increased significantly after the intervention (Table 2). Regarding differences associated with the equol producing status, all FAs analysed showed higher concentrations in the equol non-producing women, but only isovaleric acid reached statistical significance (Table 3).

## 4. Discussion

Diet modulates the composition of the intestinal microbiota [32] and, in turn, gut microbiota metabolism can determine the final metabolites produced, and thus the corresponding effects on human health. Most studies, however, have focused on the effect of fat and fiber [33,34], while dietary microcomponents, like polyphenols, have received less attention [35]. Certainly, little is known about the influence of isoflavones on the microbial populations of the gut [15,16,17,18,19].

The use of high throughput sequencing techniques allows for the determination of gut members whose culture requirements are still unknown or are uncultivable—having estimated that they are 80% of the bacterial species found by molecular tools [36]. As previously suggested, different bacteria may contribute towards equol production [9,37], but these might be present in the gut in low abundance, making their detection difficult by other techniques. In this study, with the aim of identifying changes in gut microbiota composition associated with the ingestion of isoflavones, and related to the equol-producing metabotype, we selected and made use of fecal samples from eight menopausal women receiving daily isoflavone supplementation over one month. Among these women, we selected three equol-producers and five non-producers for comparative purposes.

In the present work, the abundance of the genera *Slackia* significantly increased after the isoflavone supplementation. This genus, belonging to the family *Coriobacteriaceae*, includes described equol-producing species and strains [38,39,40] and has been associated in vivo with isoflavone metabolism [17]. Additionally, bacteria belonging to the family *Lachnospiraceae* (*Dorea* and *inc. sed.*) increased in the postmenopausal women with an equol-producing metabotype. *Lachnospiraceae inc. sed.* has previously been reported to increase significantly with isoflavone treatment in a case report of an equol-producing woman [19], while *Dorea* has been associated with isoflavone metabolism in humans in several studies [17,41]. Enrichment of some of these bacteria belonging to the *Lachnospiraceae* family, as well as *Pseudoflavonifractor*, has also been seen in in vitro fecal cultures with isoflavones [42]. The family *Lachnospiraceae* has a very large presence in the human gut and has been linked to the production of butyric acid [43], a compound with beneficial effects on the gastrointestinal epithelium [44].

Supplementation with isoflavones for one month was shown to cause a decrease in the number of species (Sobs index) as well as in the species evenness (Shannon index). These effects have previously been observed with the use of other culture-independent techniques [18]. It has been suggested that isoflavones could provide a chemical environment that selects a subset of the initial bacterial communities [17,45]. Alternatively, isoflavones might have antimicrobial effects on certain intestinal bacterial populations, as has recently been reported on pure cultures of intestinal species [46]. When considering the two different metabotypes studied, no effect in the alpha diversity indexes was observed (data not shown). However, UniFrac analysis indicated a greater similarity of the microbial communities from equol-producing women after one month of isoflavone supplementation, suggesting that isoflavones enriches the gut with microbial species involved in the degradation of isoflavones and equol production.

The production of FAs (relevant gut bacterial metabolites) was carried out to determine their relationship with the consumption of isoflavones and the production of equol. Butyric, acetic, and propionic acids are the main short-chain fatty acids (SCFAs). They are produced in the proximal colon by bacterial fermentation of non-digestible carbohydrates [47] and exert anti-inflammatory and anticarcinogenic activities [48]. In contrast, medium-chain fatty acids (MCFAs), including caproic acid (CA), by favoring TH1 and TH17 differentiation [49], could antagonize the anti-inflammatory activities of SCFAs. Branched-chain fatty acids, such as isobutyric and isovaleric acids, are often associated with protein breakdown and have been less studied.

The current data reveal an increase in CA production after isoflavone supplementation, which indicates differential microbial activity leading to the production of this compound. CA derives from chain elongation reactions in which SCFAs are converted to MCFAs mainly using ethanol or lactate as an electron donor [50]. The elongation process is mediated by microorganisms through the reverse β-oxidation pathway. Whether *Slackia*, the bacterial species found to be increased after the consumption of isoflavones in this study, produces CA is not currently known. Alternatively, isoflavone consumption could stimulate the production of CA by other intestinal microorganisms. These possibilities, however, would require further study. Although studies are still controversial, CA has been related to inflammation-regulating effects. In some studies, diminishing of the production of inflammatory cytokines by CA has been reported [51], while inflammatory effects have been reported in others [49,52].

The concentration of isovaleric acid was higher in samples from the equol non-producing group (*n* = 5). This result partially agrees with the effect of isoflavones observed previously in fecal anaerobic batch cultures [42], where isovaleric acid was reported to increase in cultures inoculated with feces from equol producers (*n* = 3). This suggests that, regardless of the equol producing status, consumption of isoflavones might stimulate the production of this FA.

In this work, although limited to the small sample size, the description of specific gut microbial and FA changes with the ingestion of isoflavones is provided, contributing to the understanding of the modulation of the gut microorganisms and their activity by these polyphenols. However, more studies with greater numbers of people, and even different populations, are needed to confirm the effects of isoflavone intake on the gut ecosystem.

## 5. Conclusions

This study allowed the changes in fecal microbial communities caused by isoflavone supplementation for one month to be monitored in a group of menopausal women. Isoflavone consumption was associated with a significant increase in the relative abundance of the genus *Slackia*, to which strains that metabolise isoflavones and produce equol are the most studied in this respect. Moreover, the taxa *Pseudoflavonifractor*, *Dorea*, and *Lachnospiraceae incertae sedis* were found in greater proportions in equol-producing women. Fecal microbial communities of equol producers were more similar to each other after isoflavone treatment, a fact that was not observed among those of equol non-producers. However, distinctive differences in the excretion of fatty acids associated with the equol status (which might be related to inflammation) were not observed.

## Figures and Tables

**Table 1:** Fecal genera showing significant greater relative abundances (% sequences) in equol-producing women before (T0) and after (T1) the soy isoflavone intervention.

		% Relative Abundance ^a^	
T0	*p*-Value ^b^	Producers (*n* = 3)	Non-Producers (*n* = 5)
*Lachnospiraceae incertae sedis*	0.025	10.34 ± 7.99	2.26 ± 1.54
**T1**			
*Dorea*	0.025	2.66 ± 1.86	0.71 ± 0.46
*Pseudoflavonifractor*	0.022	0.10 ± 0.03	0.03 ± 0.04

^a^ Mean relative abundance ± standard deviation. ^b^ Mann–Whitney test.

**Table 2:** Fecal fatty acids’ (FAs) concentration before and after the isoflavone treatment of the eight menopausal women of the study.

Time	Acetic	Propionic	Isobutyric	Butyric	Isovaleric	Valeric	Caproic *
**Basal** (*n* = 8)	20.64 ± 12.99	7.51 ± 4.75	1.73 ± 0.49	9.62 ± 6.67	2.42 ± 0.93	2.27 ± 0.74	1.24 ± 0.34
**1 month** (*n* = 8)	23.67 ± 15.87	8.44 ± 3.6	1.61 ± 0.39	12.9 ± 7.85	2.01 ± 1.12	2.55 ± 1.27	1.64 ± 1.14

Key of statistical significance: * *p* < 0.05 versus basal sample (t = 0), Wilcoxon test.

**Table 3:** Differences in fecal FAs between equol producers and non-producers after isoflavone supplementation.

Equol Status	Acetic	Propionic	Isobutyric	Butyric	Isovaleric *	Valeric	Caproic
**Producers** (*n* = 3)	19.58 ± 12.12	7.96 ± 1.97	1.46 ± 0.11	10.94 ± 5.31	1.33 ± 1.01	2.32 ± 0.42	1.14 ± 0.23
**Non-producers** (*n* = 5)	26.13 ± 17.68	8.73 ± 4.34	1.71 ± 0.47	14.08 ± 9.01	2.42 ± 1.00	2.69 ± 1.58	1.94 ± 1.36

Key of statistical significance: * *p* < 0.05, Mann–Whitney test.

## Data Availability

The raw data generated in this study can be found in the Sequence Read Archive (SRA) of the NCBI database. under accession numbers: SRR9855012-25, SRR6656999, and SRR6657000.
